# Oatmeal as a Food

**Published:** 1891-12

**Authors:** 


### OATMEAL AS A FOOD.

Oatmeal has recently received some adverse criticisms. This is not surprising, as no food article is just the thing in every case and at all times. Our daily experience convinces us of such truth by likes and dislikes of very common and most wholesome foods. It is natural and best to have some variation of diet. A thing may be just adapted to the state of the individual—bodily and mentally—while with another, it may never agree. Without entering into a lengthy and uninteresting details, chemistry, physiology and experience all prove oatmeal one of the most valuable cereal foods for producing good muscles and clear heads. But it is frequently found to disagree. Being used almost exclusively as mush, it is swallowed so easily that it is not properly mixed with the saliva—the first step for digestion. When there is little or no saliva, as in some diseases, the digestion must necessarily be very weak. A good authority says : " No saliva, no digestion." If any soft food, mush, toast, etc., is swallowed too rapidly, or any food is washed down with tea, coffee, milk, beer, wine or water, some degree of indigestion is thereby produced sooner or later, as often shown by a sense of fullness, discomfort, belching and other disturbances. If there is a lack of saliva, or that of proper quality, it is often best to eat some hard kind of bread, as thin, hard, Scotch oatmeal bread, bread crusts, rusks, etc., very slowly, and thus naturally increase the amount and quality of the saliva. Such a course is often a better and safer corrective than drugs and nostrums. Good health should be secured by correct living. The best physicians are those who recognize this fact and try to teach it to their patients. Oatmeal can be used in a variety of ways. As mush, it is often drowned in too much milk, sugar and butter, for good digestion ; is swallowed so easily that it helps lead to overeating and its bad results. Let us go slow before we reject oatmeal as a food.

